# Histone deacetylase inhibition prevents cell death induced by loss of tricellular tight junction proteins in temperature-sensitive mouse cochlear cells

**DOI:** 10.1371/journal.pone.0182291

**Published:** 2017-08-02

**Authors:** Kenichi Takano, Takuya Kakuki, Yakuto Kaneko, Takayuki Kohno, Shin Kikuchi, Tetsuo Himi, Takashi Kojima

**Affiliations:** 1 Department of Otolaryngology, Sapporo Medical University School of Medicine, Sapporo, Japan; 2 Department of Cell Science, Research Institute for Frontier Medicine, Sapporo Medical University School of Medicine, Sapporo, Japan; 3 Department of Anatomy, Sapporo Medical University School of Medicine, Sapporo, Japan; Baylor College of Medicine, UNITED STATES

## Abstract

Tricellular tight junctions (tTJs) are specialized structures that occur where the corners of three cells meet to seal adjacent intercellular space. The molecular components of tTJs include tricellulin (TRIC) and lipolysis-stimulated lipoprotein receptor (LSR) which recruits TRIC, are required for normal hearing. Although loss of TRIC causes hearing loss with degeneration of cochlear cells, the detailed mechanisms remains unclear. In the present study, by using temperature-sensitive mouse cochlear cells, US/VOT-E36 cell line, we investigated the changes of TRIC and LSR during cochlear cell differentiation and the effects of histone deacetylase (HDAC) inhibitors against cell degeneration induced by loss of TRIC and LSR. During cell differentiation induced by the temperature change, expression of TRIC and LSR were clearly induced. Treatment with metformin enhanced expression TRIC and LSR via AMPK during cell differentiation. Loss of TRIC and LSR by the siRNAs induced cell death in differentiated cells. Treatment with HDAC inhibitors trichostatin A and HDAC6 inhibitor prevented the cell death induced by loss of TRIC and LSR. Collectively, these findings suggest that both tTJ proteins TRIC and LSR have crucial roles for the differentiated cochlear cell survival, and that HDAC inhibitors may be potential therapeutic agents to prevent hearing loss.

## Introduction

The tight junctions (TJs) between epithelial cells are necessary to maintain cell polarity and the transepithelial barrier, and regulate the flow of solutes through paracellular spaces [1, 2]. In the inner ear, TJs between epithelial cell that line the cochlear duct (or scala media) function to compartmentalize endolymph and perilymph [3]. Tricellular tight junctions (tTJs) occur at the convergence between two bicellular TJs, and aid in the formation of a strong barrier for the cellular sheet [4]. The formation of tricellular contacts requires tricellulin (TRIC), the first protein identified at these contacts [4], and the newly identified lipolysis-stimulated lipoprotein receptor (LSR) [5]. In particular, the LSR localizes at the corners of epithelial cells to generate a landmark for tricellular tight junction formation, while TRIC is recruited to the tricellular contacts via its interaction with LSR [5].

Previous reports demonstrate that knockdown of occludin causes TRIC to mislocalize to bicellular TJs, resulting in progressive cochlear hair cell apoptosis [6–8]. Mutations in the gene encoding TRIC lead to autosomal recessive nonsyndromic hearing loss (DFNB49) [9, 10]. In comparison, LSR has two closely related proteins encoded in the mammalian genome, immunoglobulin-like domain-containing receptor (ILDR) 1 and ILDR2. ILDR1 is the causative gene of familial nonsyndromic deafness (DFNB42) and mediates TRIC recruitment, which is required for normal hearing [11, 12].

Metformin is an antidiabetic drug known to protect against cisplatin-induced ototoxicity [13] and gentamycin-induced apoptosis in auditory cells [14]. Similarly, histone deacetylase inhibitors (iHDACs) reportedly limit noise-induced outer hair cells death and hearing loss [15, 16], and attenuate gentamicin-induced hearing loss [17]. HDACs are a class of enzymes that remove acetyl groups from the lysine residues of target proteins, thereby promoting chromatin condensation and reduced transcription [18]. Eighteen mammalian HDACs have been identified to dates and are divided into 4 classes: class I HDACs (HDACs 1, 2, 3, and 8), class II HDACs (HDACs 4, 5, 6, 7, 9, and 10), class IV (HDAC 11), and class III (sirtuin family: SIRT1-SIRT7) [19]. Class II HDACs are further divided into two subgroups: Class IIa (4, 5, 7, and 9) and IIb (6 and 10). In particular, HDAC6 is a unique cytoplasmic enzyme that regulates many biological processes via its deacetylase and ubiquitin-binding activities. For example, HDAC6 is a target for protection and regeneration following nervous system injury [20]. Moreover, HDAC6 is a crucial driver for the disassembly of cilia in sensory hair cells of the mammalian cochlea, which play important role in maintaining normal hearing [21]. On the other hand, previous studies described that metformin elevates LSR expression in human endometrial cancer cells [22], whereas iHDACs upregulate TJ molecules in cancer cells [23, 24]. However, the functional significance of tTJ molecules, including LSR and TRIC, and their association with HDACs in cochlear cell death remains unclear.

In the present study, we investigated changes in tTJs during differentiation using temperature-sensitive mouse cochlear cells, as well as the effect of metformin and iHDACs on LSR and TRIC expression. In addition, we examined the correlation between the presence of tTJs and apoptotic cochlear cell death, and the potential protective properties of iHDACs against cochlear cell death in an auditory cell line.

## Materials and methods

### Reagents and antibodies

Metformin was purchased from Wako (Tokyo, Japan). Trichostatin A (TSA) was purchased from Sigma-Aldrich (St. Louis, MO, USA). HDAC6 inhibitor was purchased from Santa Cruz Biotechnology (Dallas, TX, USA). Rabbit polyclonal anti-LSR antibodies were obtained from Novus Biologicals (Littleton, CO, USA). Rabbit polyclonal anti-TRIC and ZO-1 antibodies were obtained from Zymed Laboratories (San Francisco, CA, USA). Rabbit polyclonal anti-acetylated lysine, anti-phospho-AMPKα (Thr172), and anti-HDAC6 antibodies were obtained from Cell Signaling Technology (Danvers, MA, USA). Mouse monoclonal anti-acetylated tubulin and polyclonal rabbit anti-actin antibodies were obtained from Sigma-Aldrich. Monoclonal mouse anti-α-tubulin and anti-β-tubulin antibodies were purchased from Wako. Alexa 488 (green)-conjugated anti-rabbit IgG and Alexa 594 (red)-conjugated anti-mouse IgG antibodies were acquired from Molecular Probes, Inc. (Eugene, OR, USA). The ECL Western blot system was purchased from GE Healthcare UK, Ltd. (Buckinghamshire, UK).

### Temperature-sensitive mouse cochlear precursor cells

We used the conditionally immortal cell line University of Sheffield/ventral otocyst-epithelial cell line clone 36 (US/VOT-E36) gifted from Professor Matthew Holley [25]. In this model, cell proliferation is maintained when cultured at 33°C, but undergo growth arrest and differentiation at 39°C. The cells were cultured in minimum essential medium (Nacalai Tesque, Inc., Kyoto, Japan) supplemented with 10% fetal bovine serum (FBS, Invitrogen; Carlsbad, CA, USA), 100 μg/mL streptomycin, and 50 μg/mL amphotericin B. The cells were plated on 60-mm culture dishes (Corning Glass Works, Corning, NY, USA) were coated with rat tail collagen (500 μg dried tendon/mL in 0.1% acetic acid) in a humidified atmosphere with 5% CO2 at 33°C. After incubation at 33°C for several days, the temperature was raised to 39°C to induce cell differentiation. For some experiments, cells were treated with the indicated concentrations of metformin, TSA, or an HDAC6 inhibitor for 24 h prior to analysis.

### RNA interference and transfection

Duplex siRNA oligonucleotides directed to TRIC and LSR were synthesized by Santa Cruz Biotechnology. The sequences were as follows: TRIC sense: 5′-CGAUCGAGAACGCUAUAAGtt-3′, antisense: 5′-CUUAUAGCGUUCUCGAUCGtt-3′; LSR sense: 5′-CAUGAGGGUCCUAUACUAUtt-3′; antisense: 5′-AUAGUAUAGGACCCUCAUGtt-3′. Cells were transfected with LipofectamineTM RNAiMAX Reagent (Invitrogen) at 24 h after plating. A scrambled siRNA sequence (BLOCK-iT Alexa Fluor fluorescent, Invitrogen) was employed as control siRNA.

### RNA isolation and RT-PCR

Total RNA was extracted and purified using TRIzol (Invitrogen). Total RNA (1 μg) was reverse-transcribed into cDNA using a mixture of oligo (dT) and Superscript II reverse transcriptase under the recommended conditions (Invitrogen) in a total volume of 20 μL for 50 min at 42°C, and terminated by a 15 min incubation at 70°C. PCR was performed in a 20-μL mixture containing 100 pM primer pairs, 1.0 μL cDNA, PCR buffer, dNTPs, and Taq DNA polymerase under the recommended conditions (Takara, Kyoto, Japan). Amplifications were carried out for 35 or 40 cycles depending on the target gene with the following thermocycler protocol: 15 s at 96°C, 30 s at 55°C or 57°C, and 60 s at 72°C. The final elongation time was 7 min at 72°C. Then, 6 μL of PCR product was analyzed by 1% agarose gel electrophoresis with ethidium bromide staining with a GeneRuler 100-bp DNA ladder (Fermentas, Ontario, Canada). The PCR primers were as follows: mouse TRIC sense: 5′-CTCGGAGACATCGGGAGTTC-3′, antisense: 5′-GCTGATCCCTCTGTCGATCACT-3′; mouse LSR sense: 5′-CGCAGAGCTCATTGTCCTTTGATTG-3′, antisense: 5′-GGAGGTTACTTCACTTCACTCATGGCCCG-3′; G3PDH sense 5′-ACCACAGTCCATGCCATCAC-3′, antisense 5′-TCCACCACCCTGTTGCTGTA-3′.

### Western blot analysis

Cultured cells were scraped from a 60-mm dish containing 300 μL of buffer (1 mM NaHCO3 and 2 mM phenylmethylsulfonyl fluoride), collected in microcentrifuge tubes, and then sonicated for 10 seconds. The protein concentrations of the samples were determined using a BCA protein assay regent kit (Pierce Chemical Co., Rockford, IL, USA). Proteins (15 μg/lane) were separated by electrophoresis in 5–20% SDS polyacrylamide gels (Wako, Osaka, Japan), and transferred to nitrocellulose membranes (Immobilon; Millipore Co.; Bedford, UK). The membrane were incubated for 30 min at room temperature with blocking buffer (25 mM Tris, pH 8.0, 125 mM NaCl, 0.1% Tween 20, and 4% skim milk), and then the appropriate primary antibody (1:1000 dilution) for 1 h. The probed membranes were incubated with HRP-conjugated anti-mouse or anti-rabbit IgG antibodies at room temperature for 1 h and the immunoreactive bands detected with an ECL Western blot system.

### Immunoprecipitation

Cultured cells were scraped from a 60-mm dish containing 600 μL NP-40 lysis buffer (50 mM Tris—HCl, 1% NP-40, 0.25 mM Na-deoxycholate, 150 mM NaCl, 2 mM EGTA, 0.1 mM Na3VO4, 10 mM NaF, 1 mM PMSF), collected in microcentrifuge tubes, and then sonicated for 10 s. The lysates were incubated with protein A-Sepharose CL-4B (Pharmacia LKB Biotechnology, Uppsala, Sweden) for 30 min at 4°C and then clarified by centrifugation at 5,000 rpm for 3 min at 4°C. The supernatants were incubated with polyclonal anti-LSR, anti-TRIC, and anti-acetylated lysine antibodies bound to protein A-Sepharose CL-4B overnight at 4°C. After incubation, immunoprecipitates were washed extensively with the same lysis buffer and analyzed by western blotting with the indicated antibodies (1:1000 dilution).

### Immunocytochemistry

Cells cultured in a 35-mm glass-coated dish (Iwaki, Chiba, Japan) were fixed with cold acetone and ethanol (1:1) at -20°C for 10 min, rinsed in PBS, and then incubated with anti-acetylated tubulin (1:400), anti-TRIC (1:200), anti-LSR (1:200), or anti-ZO-1 (1:200) at room temperature for 1 h. Alexa Fluor 488 (green)-conjugated anti-rabbit IgG and Alexa Fluor 594 (red)-conjugated anti-mouse IgG (Invitrogen) were used as secondary antibodies. Specimens were examined with either an epifluorescence (Olympus, Tokyo, Japan) or confocal laser scanning microscope (LSM5; Carl Zeiss, Jena, Germany).

### Scanning electron microscopy (SEM)

For scanning electron microscopy (SEM), cells cultured on 15-mm cover glasses (Matsunami Glass, Ind., Ltd, Bellingham, WA, USA) were fixed in 2.5% glutaraldehyde in 0.1 M PBS (pH 7.3) at 4°C for 3 h. After rinsing in PBS, the cells were postfixed in 1% osmium tetroxide in PBS at 4°C for 3 h, dehydrated with a graded ethanol series (70% to 100% ethanol) at 4°C for 20 min in each step, and dried with butyl alcohol. The dried samples were sputter-coated with platinum and examined under a scanning electron microscope (S-4300; Hitachi, Tokyo, Japan).

### Transepithelial electrical resistance (TER) analysis

Cells were cultured to confluence on the inner chambers of 12-mm Transwell inserts with 0.4-μm pore filters (Corning Life Sciences, NY, USA). TER was measured using an EVOM voltmeter with an ENDOHM-12 (World Precision Instruments, Sarasota, FL, USA). Data are expressed in Ω/cm2 and presented as the mean ± S.D. of triplicate experiments. For calculation, the resistance of blank filters was subtracted from that of filters covered with cells.

### Apoptosis and necrosis assay

Apoptosis and necrosis assay were examined with a FAM-FLICA in vitro Caspase Detection Kit (ImmunoChemistry Technologies, Bloomington, IN, USA). Briefly, cultured cells were seeded onto 35-mm glass-coated wells (Iwaki, Chiba, Japan), incubated with FLICA at 39°C for 2 h, replated in fresh media, and then incubated again at 39°C for 1 h. The stained cells were incubated with Hoechst33258 and propidium iodide at room temperature for several minutes, fixed, and visualized with an epifluorescence microscope (Olympus, Tokyo, Japan).

### Statistical analysis

All data are representative of at least three independent experiments. Results are given as the means ± SEM. Differences between groups were tested by analysis of variance (ANOVA) followed by a post-hoc test, or unpaired two-tailed Student’s t test. P < 0.05 was considered to be statistically significant.

## Results

### Metformin induces expression of TRIC and LSR via AMPK activation during cochlear cell differentiation

**Fig 1:**
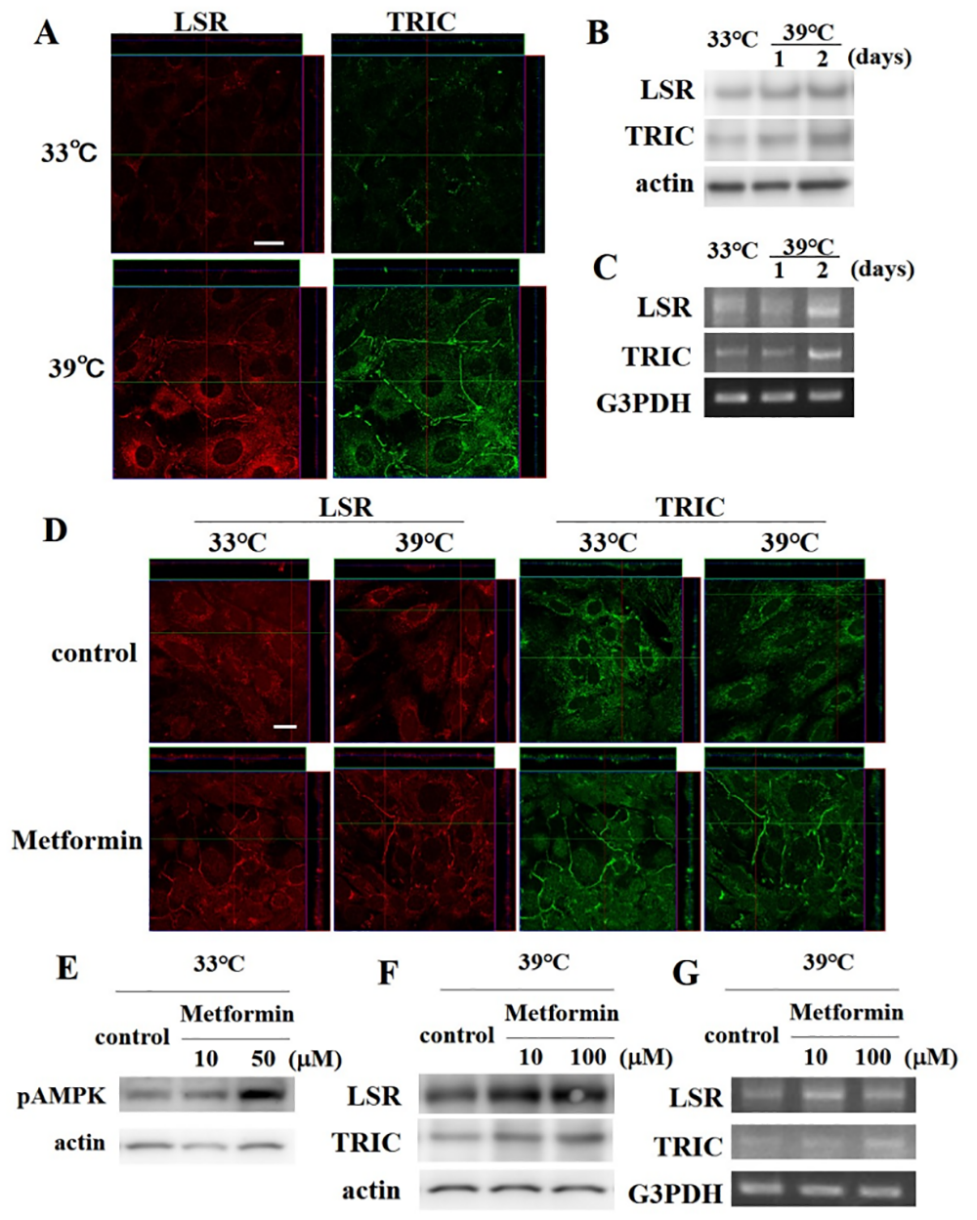
Induced LSR and TRIC expression in temperature-sensitive mouse cochlear cells. (A) LSR and TRIC immunocytochemistry of cells incubated at 33°C and 39°C. Scale bar: 20 μm. (B) Western blotting and (C) RT-PCR analysis of LSR and TRIC expression after 1 and 2 days incubated at 39°C. (D) LSR and TRIC immunocytochemistry of cells incubated at 33°C and 39°C after treatment with 100 μM metformin. Scale bar: 20 μm. (E) Western blotting for phospho-AMP kinase (pAMPK) in cells cultured at 33°C treated with 10 or 50 μM metformin. (F) Western blotting and (G) RT-PCR for LSR and TRIC expression in cells incubated at 39°C treated with 10 or 100 μM metformin.

To investigate changes in TRIC and LSR expression during cochlear cell differentiation, temperature-sensitive mouse cochlear cells were examined by immunocytochemistry, western blotting, and RT-PCR analysis. Few TRIC molecules and LSRs were observed in the cytoplasm of undifferentiated cochlear cells; however, abundant TRIC molecules and LSRs were observed on the membrane and in the cytoplasm in differentiated counterparts (Fig 1A). Consistently, TRIC and LSR expression increased with progressive differentiation in western blotting and RT-PCR analyses (Fig 1B).

To investigate whether metformin induced TRIC and LSR expression, the cochlear cells were pretreated with metformin and then examined by immunocytochemistry. Notably, both undifferentiated and differentiated cochlear cells showed increased TRIC and LSR expression in the presence of metformin (Fig 1D). Moreover, western blotting showed elevated AMPK phosphorylation following treatment with 50 μM metformin (Fig 1E), as well as a dose-dependent increase in TRIC and LSR expression at the protein (Fig 1F) and mRNA level (Fig 1G).

### HDAC inhibitors induce expression of TRIC, LSR and tubulin acetylation, and increased epithelial barrier function

**Fig 2:**
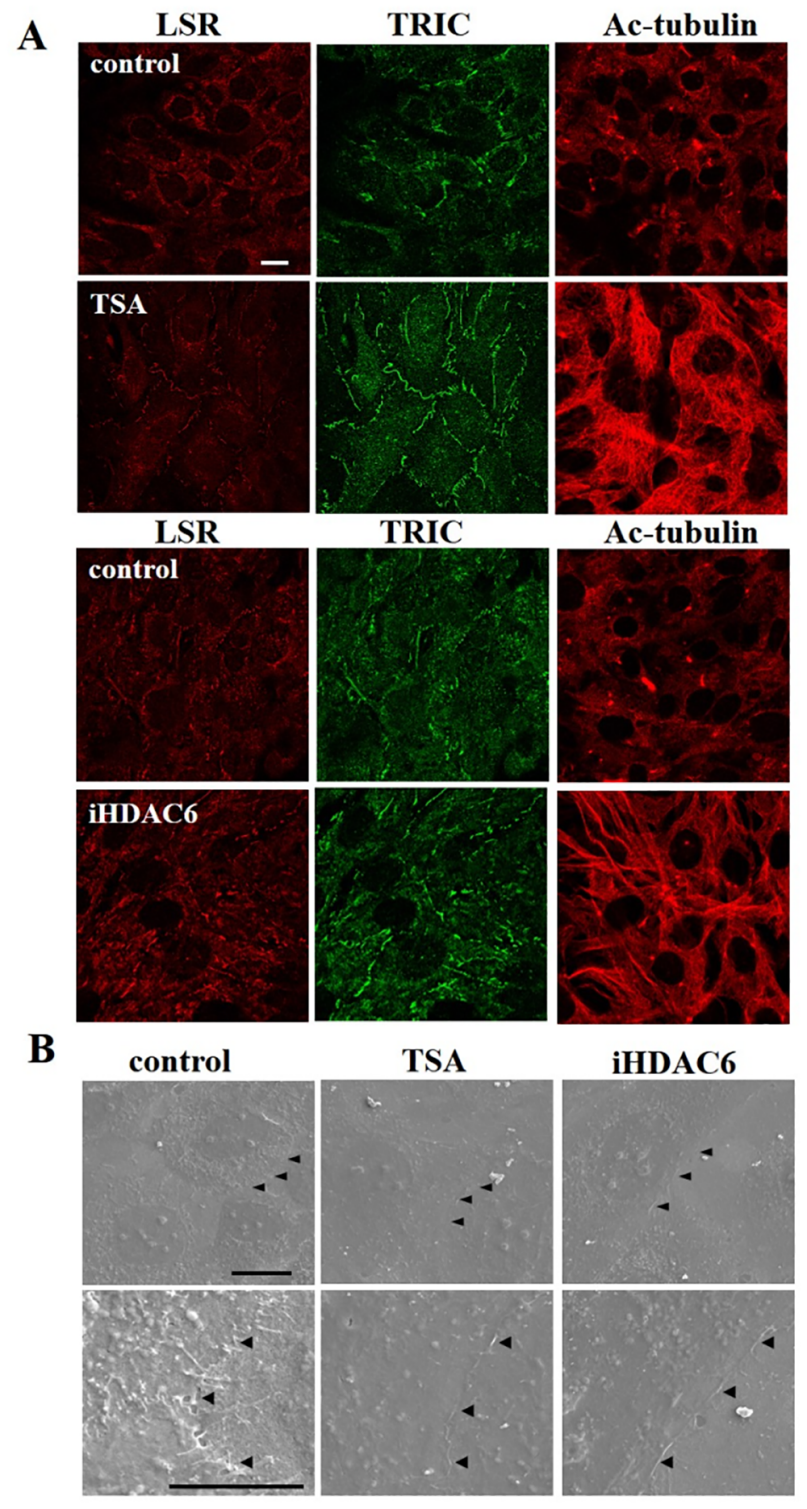
Trichostatin A (TSA) and histone deacetylase 6 inhibitor (iHDAC6) treatment induced LSR, TRIC, and acetylated-tubulin (Ac-tubulin) expression in differentiated cochlear cells. (A) Immunocytochemical staining for LSR, TRIC, and Ac-tubulin after TSA and iHDAC6 treatment. Scale bar: 20 μm. (B) Scanning electron microscopy (SEM) of differentiated cochlear cells after treatment with TSA and iHDAC6. Arrow heads indicate the sealing elements between two adjacent cells. Scale bars: 20 μm.

**Fig 3:**
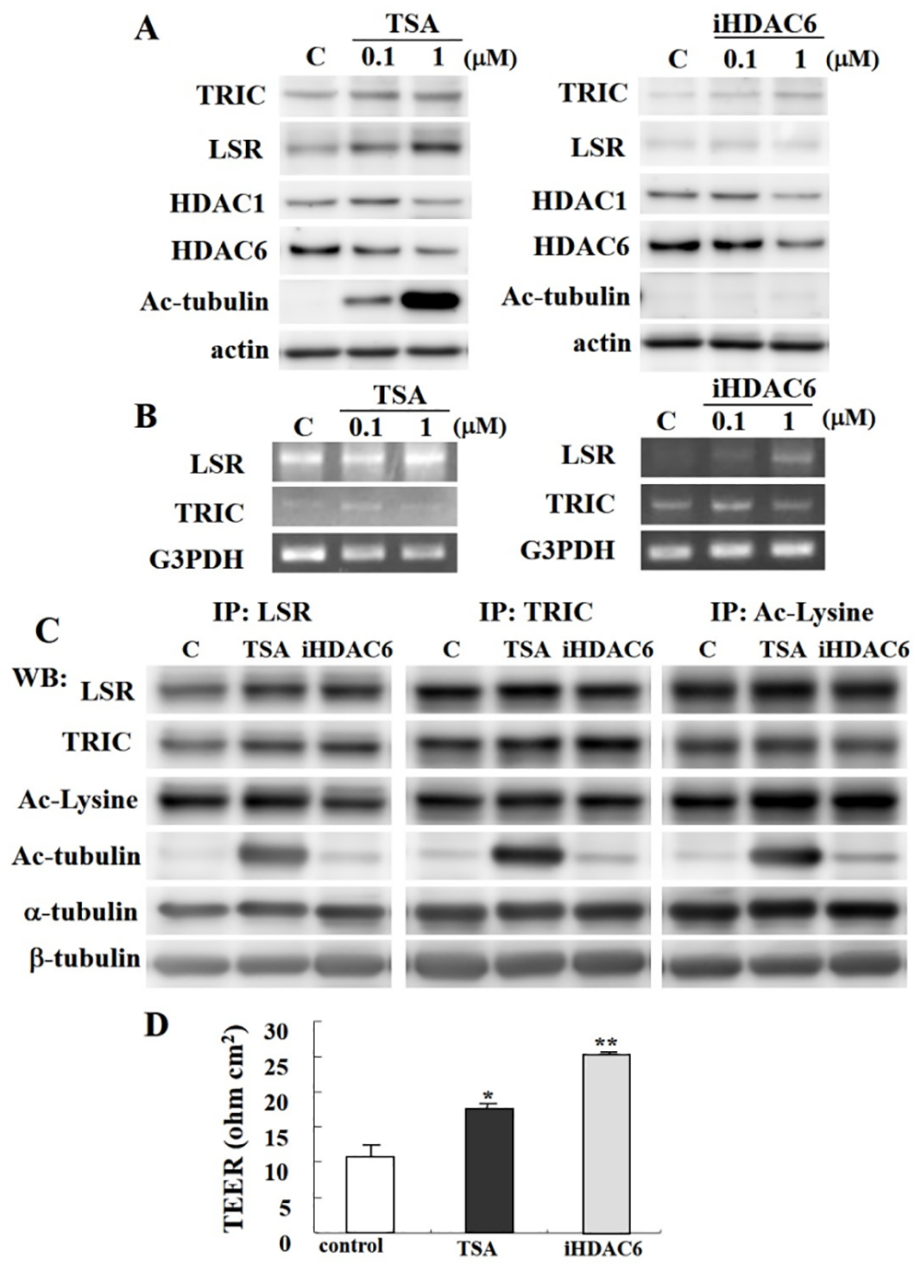
(A) Western blotting for TRIC, LSR, HDAC1, HDAC6, and Ac-tubulin in differentiated cochlear cells after treatment with 0.1 or 1 μM TSA and 0.1 or 1 μM iHDAC6. (B) RT-PCR for LSR and TRIC after treatment with 0.1 or 1 μM TSA and 0.1 or 1 μM iHDAC6. (C) Western blotting for LSR, TRIC, acetylated lysine (Ac-Lysine), Ac-tubulin, α-tubulin, β-tubulin in LSR, TRIC, or Ac-Lysine immunoprecipitates (IP) from cochlear cells treated with 1 μM TSA and 1 μM iHDAC6. (D) Transendothelial resistance (TER) analysis in cochlear cells treated with 1 μM TSA and 1 μM iHDAC6. *P < 0.05 and **P < 0.01 vs. control.

To examine the influence of TRIC, LSR, and acetylated tubulin (Ac-tubulin) in differentiated cochlear cells, we used trichostatin A (TSA) and a HDAC6 inhibitor (iHDAC6). Immunocytochemical analysis revealed increased TRIC and LSR expression at the periphery of differentiated cochlear cells treated with TSA and iHDAC6 (Fig 2A). Ac-tubulin was also increased in cells treated with TSA and iHDAC6 (Fig 2A). Similarly, scanning electron microscopy (SEM) analysis showed loose sealing elements like zipper between adjacent control cells, but tight sealed junctions such as line in cells treated with TSA and iHDAC6 (Fig 2B). Consistent with these findings, western blotting and RT-PCR analysis demonstrated that TRIC and LSR expression increased with TSA and iHDAC6 treatment in a dose dependent manner, whereas the expression of HDAC1 and HDAC6 were decreased (Fig 3A and 3B). Moreover, increased Ac-tubulin was observed in treated cells, particularly in those treated with TSA.

The functional significance of TRIC and LSR expression in differentiated cochlear cells after TSA or iHDAC6 treatment was examined by immunoprecipitation using anti-TRIC, anti-LSR, and anti-acetyl-Lysine (Ac-Lysine) antibodies (Fig 3C). The expression of TRIC and LSR in cells treated with TSA or iHDAC6 was found to be increased, whereas the expression of Ac-Lysine was not changed. An increased in Ac-tubulin was also found in cells treated with TSA or iHDAC6; in particular, TSA induced stronger expression of Ac-tubulin. However, there was no change in the expression of α- and β-tubulin.

Subsequently, TER was assessed to determine whether the increase in tTJs observed following iHDAC treatment altered epithelial barrier function. As expected, cochlear cells treated with TSA or iHDAC6 displayed a significant increase in TER value (Fig 3D).

### HDAC inhibitors do not alter ZO-1 expression

**Fig 4:**
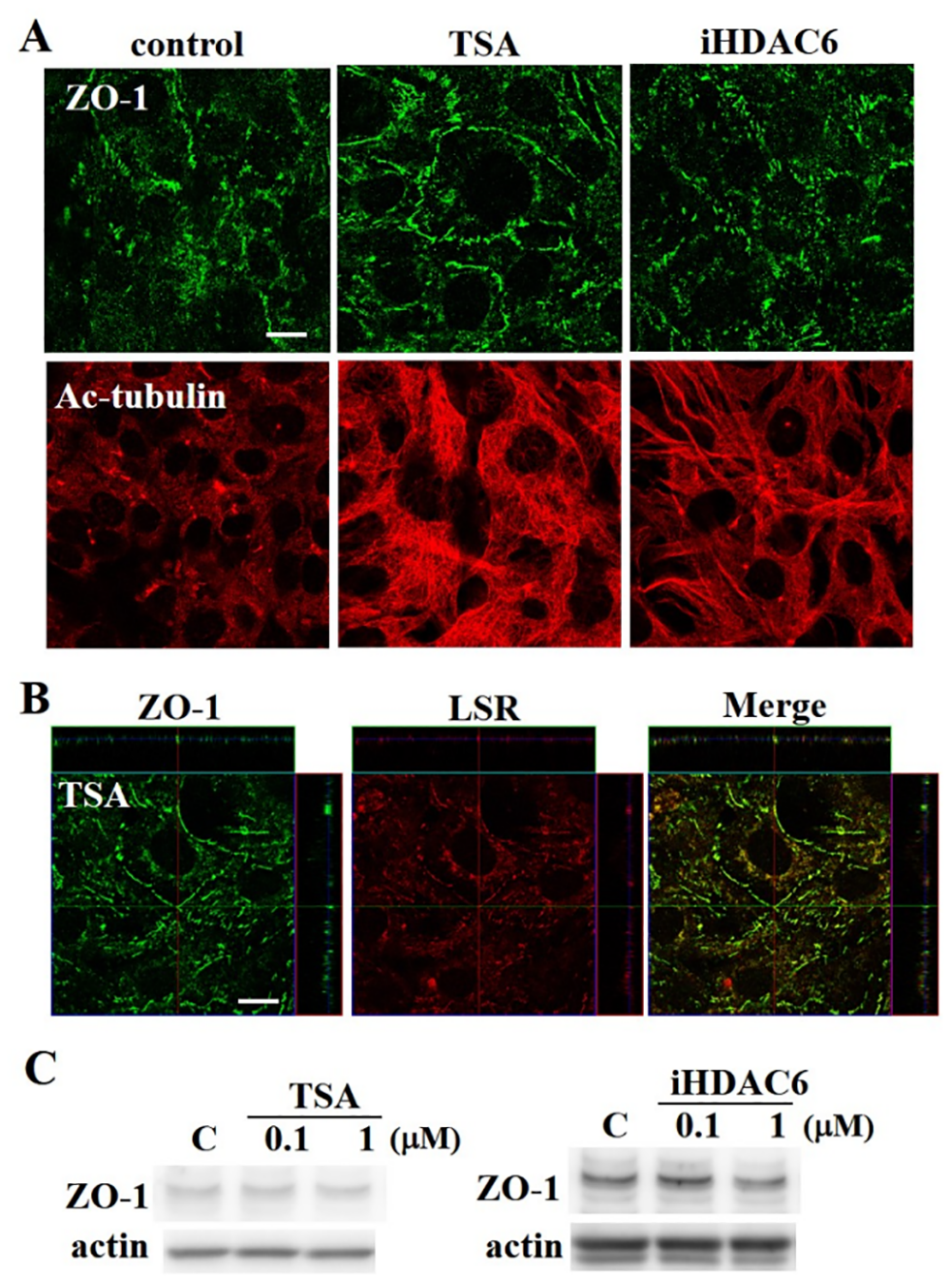
ZO-1 expression was unchanged following treatment with TSA and iHDAC6. (A) Immunocytochemical staining for ZO-1, LSR, TRIC, and Ac-tubulin in differentiated cochlear cells after TSA and iHDAC6 treatment. Scale bar: 20 μm. (B) Double-immunocytochemistry for ZO-1 and LSR in differentiated hair cells after TSA. Scale bar: 20 μm. (C) Western blotting for ZO-1 after treatment with 0.1 or 1 μM TSA and 0.1 or 1 μM iHDAC6.

Immunostaining and western blotting were used to examine whether HDAC inhibitors altered a bicellular tight junction protein ZO-1. No changes in ZO-1 expression were observed with either treatment (Fig 4A and 4C). Moreover, ZO-1 was in part colocalize with LSR in cells treated with TSA (Fig 4B).

### Loss of TRIC and LSR induce cochlear cell death during cochlear cell differentiation

**Fig 5:**
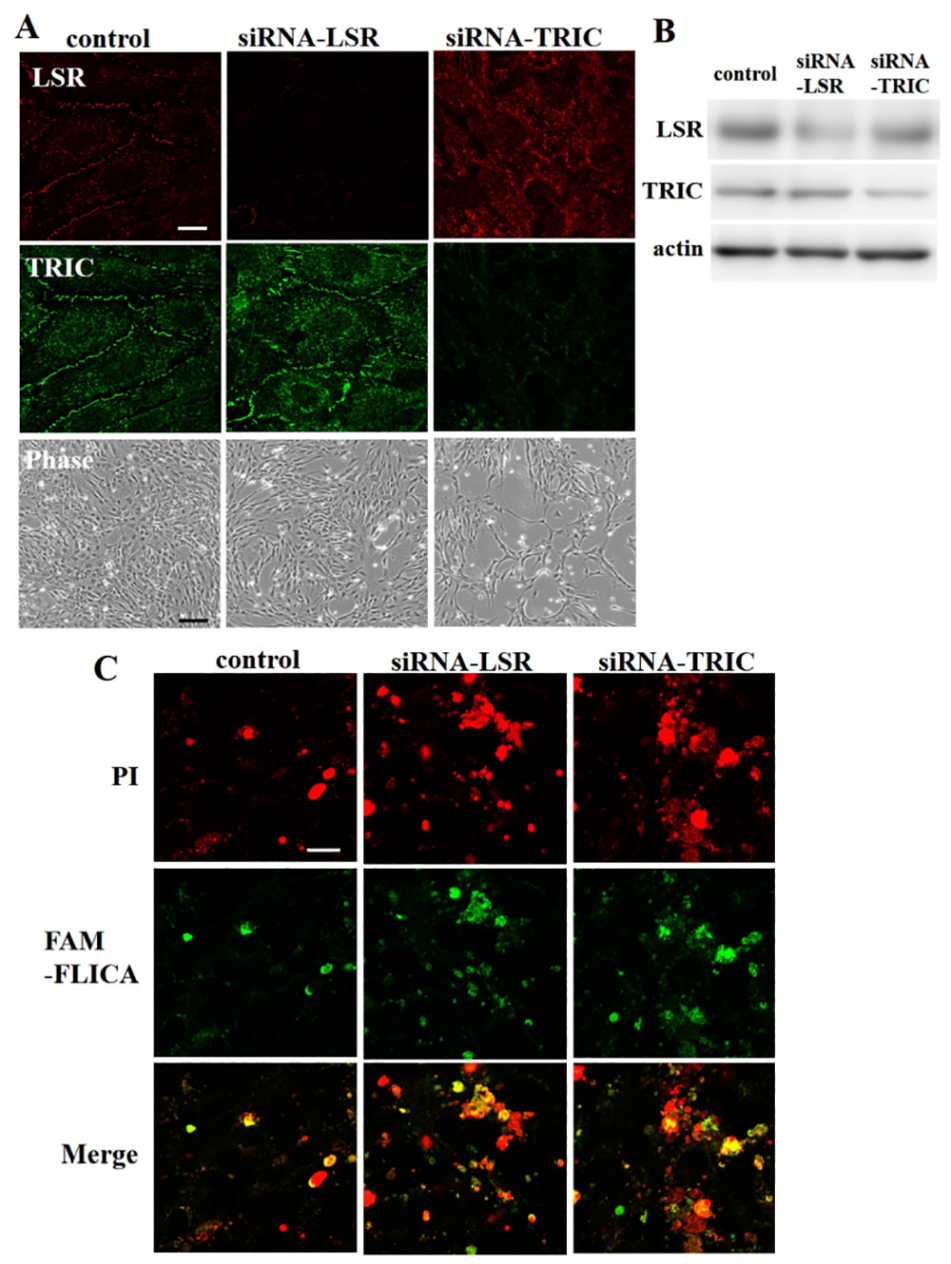
(A) Immunocytochemistry and (B) western blotting for LSR and TRIC in siRNA-knockdown cochlear cells. White scale bar: 20 μm. Black scal bar: 150μm. (C) Cell death analysis by fluorescence microscopy for propidium iodide (PI) and caspase staining. Scale bar: 20 μm.

TRIC and LSR siRNA knockdown cells were used to investigate the role of each factor in differentiated cochlear cells. As expected, immunostaining and western blotting analysis demonstrated an effective knockdown in transfected cells (Fig 5A and 5B). Interestingly, phase contrast imaging also showed that siRNA-knockdown cells displayed a suppressed viability as compared to untransfected counterparts. As such, we analyzed cell death by FLICA and PI staining to determine whether the decrease in cell number resulted from increased cell death or attenuated cell growth. Cell imaging showed that PI- and FLICA colocalized in both LSR and TRIC siRNA-knockdown cells (Fig 5C).

**Fig 6:**
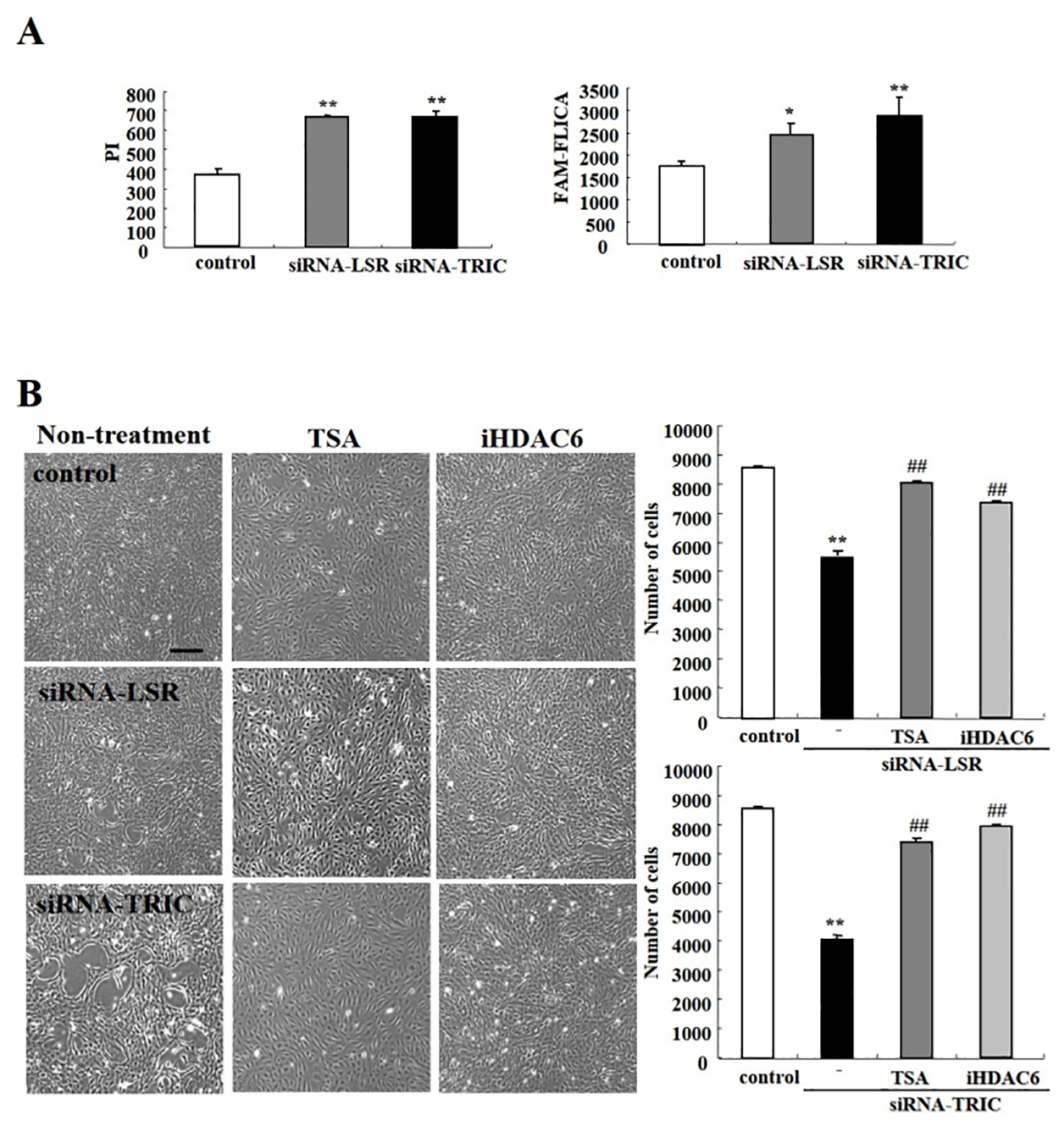
LSR and TRIC knockdown induced cochlear cell death. (A) Hoechst H33342/PI/FLICA viability analysis in cochlear cells transfected with LSR or TRIC siRNA. Graphs show the number of cochlear cells with positive PI or caspase staining. *P < 0.05 and **P < 0.01 vs. control. (B) Phase contrast microscopy images and the number of siRNA-transfected cells after treatment with TSA and iHDAC6. Scale bar: 150 μm.

We next examined number of PI and FLICA double-positive cells after TRIC or LSR knockdown. Notably, PI staining increased significantly after knockdown of LSR and TRIC (Fig 6A and S6 Fig), whereas FLICA staining increased only in TIRC knockdown cells (Fig 6A and S6 Fig). Furthermore, TSA and iHDAC6 treatment greatly increased the viability of LSR and TRIC knockdown cells (Fig 6B).

### HDAC inhibitors prevent cochlear cell death induced by loss of TRIC and LSR during cochlear cell differentiation

**Fig 7:**
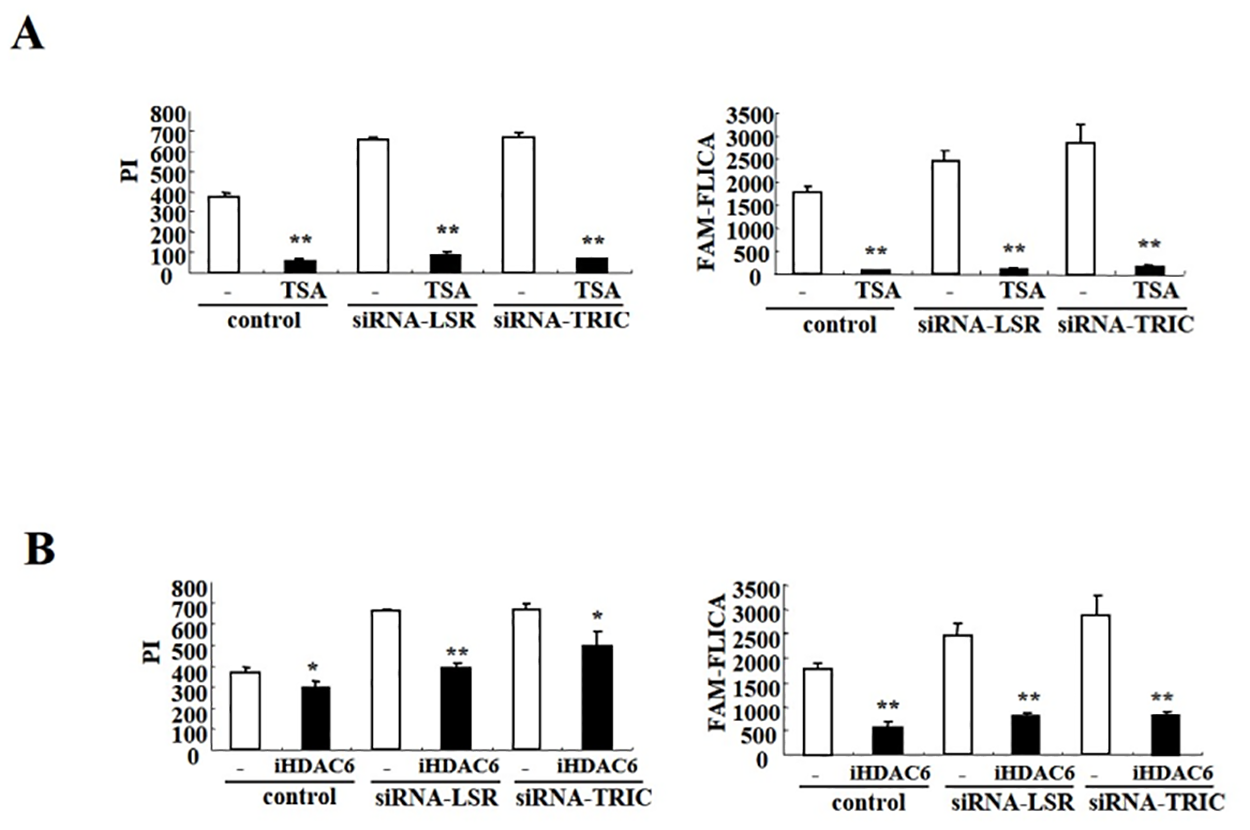
TSA and iHDAC6 treatment blocked apoptotic and necrotic cochlear cell death induced by LSR and TRIC knockdown. (A) Hoechst H33342/PI/FLICA viability analysis in cochlear cells transfected with LSR and TRIC siRNA and treated with (A) TSA or (B) iHDAC6. Graphs show the number of treated cells positive for PI or FLICA staining. *P < 0.05 and **P < 0.01 vs. control.

To confirm that iHDACs were sufficient in rescuing cochlear cell apoptosis and necrosis, we examined the viability of LSR and TRIC knockdown cells following TSA treatment. Significantly, PI and FLICA staining was markedly reduced in knockdown cells following treatment with TSA, as well control cells (Fig 7A and S7 Fig). Moreover, a significant decrease was found in PI- or FLICA-positive LSR and TRIC knockdown cells following iHDAC6 treatment, albeit to a less extent than that observed with TSA (Fig 7B and S8 Fig).

## Discussion

The present study demonstrated that metformin and iHDACs induced the expression of TRIC and LSR, but not of ZO-1, and increased epithelial barrier function in differentiated cochlear cells. We also found that knockdown of TRIC and LSR led to cochlear cell death via apoptosis and necrosis, which could be mitigated by treatment with either TSA and iHDAC6.

The barrier function of tight junctions in cochlear cells is critical to separate high K^+^/low Na^+^ endolymph from low K^+^/high Na^+^ perilymph. TRIC and LSR are essential for the generation of tTJs necessary for full barrier function of epithelial cellular sheets. In this study, we used temperature-sensitive mouse cochlear precursor cells to investigate the behavior of tTJ proteins during the sensory cochlear cell differentiation. Notably, TRIC and LSR expression were increased in differentiated cells in an AMPK-dependent manner. Consistently, treatment with metformin, a type 2 diabetes drug that elevates AMPK activity, enhanced TRIC and LSR expression in both undifferentiated and differentiated cochlear cells.

iHDACs are also known to reduced noise-, gentamicin-, and cisplatin-induced ototoxicity [15, 16, 17, 26]; however, the mechanism by which this occurs remains unclear. In the present study, iHDAC treatment increased both TRIC and LSR expression. Mutations in the gene encoding TRIC are responsible for autosomal recessive nonsyndromic hearing loss [9, 10], which was determined to result from a loss in mechanosensory cochlear cells in transgenic mouse studies [27]. Significantly, disruption of the strands of intramembrane particles connecting bicellular and tricellular junctions may affect the paracellular permeability of ions or small molecules, creating a toxic microenvironment for cochlear cells [26]. On the other hand, recent report indicates that LSR regulates TRIC recruitment to tTJs [5]. Consistently, LSR has two homologous genes known as ILDR1 and ILDR2, of which mutations in ILDR1 result in familial nonsyndromic deafness [11].

Moreover, the siRNA knockdown of either TRIC or LSR resulted in differentiated cochlear cell death via apoptosis and necrosis. Recently, Kamitani et al. reported that *TRIC*-knockout mice displayed hearing loss associated with increased cochlear cell apoptosis [28], resulting in increased paracellular ion permeability in the organ of Corti. Notably, exposure to endolymph (with high K^+^ concentration) likely causes prolonged cochlear hair cell depolarization and subsequent cell death. In addition, increased paracellular permeability has been observed in TRIC knockdown Eph4 cells4, while the opposite was found in MDCK II cells with TRIC overexpression [29]. Thus, disruption of tTJs and epithelial barrier function likely alters ion homeostasis, leading to cochlear cell death; however, we cannot exclude the possibility that knockdown of tTJ molecules may affect the survival of cochlear cell directly.

Lastly, we showed that iHDACs prevent cochlear cell death induced by TRIC and LSR knockdown. HDACs have a crucial role in both, transcription regulation and protein modification [30]. Previous studies have identified iHDACs as a promising strategy for therapeutic intervention in cancer, neurological disorders, immune disorders31, and hearing loss [15, 16, 17, 26]. The deacetylation of histone core proteins may trigger cell death; thus, iHDACs could function to attenuate the apoptotic-signaling pathways involved in noise-induced hearing loss [15, 16]. Although the precise mechanisms relating iHDACs to cochlear cell survival remain unclear, our results suggest an important role for iHDAC in increased tTJs and epithelial barrier function, thereby mitigating cochlear cell death.

The present study used TSA and iHDAC6 as iHDACs. TSA is an anti-inflammatory agent derived from streptavidin metabolites and reduces cisplatin-induced ototoxicity by regulating the IL-4/STAT6 signaling pathway [26]. In addition, TSA leads to a prolonged NF-κB activation and a subsequent increase in the pro-inflammatory response [31]. Tight junction proteins are regulated by various cytokines and growth factors via distinct signal transduction pathways [32]. JNK and NF-κB are largely involved in the regulation of tTJs, including TRIC expression [33]. On the other hand, HDAC6 is unique among the classical HDAC family in being a cytoplasmic enzyme that regulates several important biological processes, including cell migration, immune synapse formation, viral infection, and misfolded protein degradation [34]. Additionally, HDAC6 was recently identified as a target for protection and regeneration following nervous system injury20. Moreover, iHDAC6 induce Ac-tubulin and prevent the loss of intestinal TJ proteins and promote cellular viability during hemorrhagic shock and anoxia [35]. Thus, TSA and iHDAC6 may increase the number of tTJs to prevent cochlear cell death.

## Conclusions

The results of the present study provide novel evidence that TRIC and LSR are tTJ constituent proteins upregulated in response to metformin and iHDAC treatment and necessary for the survival of differentiated mouse cochlear cells. Collectively, these data support the novel application of metformin and iHDACs as pharmacotherapy for various forms of ototoxicity.

## Supporting information

S1 Fig Graph of Fig 1 (B) western blotting and (C) RT-PCR analysis of LSR and TRIC expression after 1 and 2 days incubated at 39°C. (TIF)

S2 Fig Graph of Fig 1 (E) western blotting for phospho-AMP kinase (pAMPK) in cells cultured at 33°C treated with 10 or 50 μM metformin. (F) Western blotting and (G) RT-PCR for LSR and TRIC expression in cells incubated at 39°C treated with 10 or 100 μM metformin.
(TIF)

S3 Fig Graph of Fig 3 (A) western blotting for TRIC, LSR, HDAC1, HDAC6, and Ac-tubulin in differentiated cochlear cells after treatment with 0.1 or 1 μM TSA and 0.1 or 1 μM iHDAC6. (TIF)

S4 Fig Graph of Fig 3 (B) RT-PCR for LSR and TRIC after treatment with 0.1 or 1 μM TSA and 0.1 or 1 μM iHDAC6. (TIF)

S5 Fig Graph of Fig 4 (C) western blotting for ZO-1 after treatment with 0.1 or 1 μM TSA and 0.1 or 1 μM iHDAC6, and Fig 5 (B) western blotting for LSR and TRIC in siRNA-knockdown cochlear cells. (TIF)

S6 Fig Images of Hoechst H33342/PI/FLICA viability analysis in cochlear cells transfected with LSR or TRIC siRNA. Scale bar: 20 μm.
(TIF)

S7 Fig Images of Hoechst H33342/PI/FLICA viability analysis in cochlear cells transfected with LSR and TRIC siRNA and treated with TSA. Scale bar: 20 μm.
(TIF)

S8 Fig Images of Hoechst H33342/PI/FLICA viability analysis in cochlear cells transfected with LSR and TRIC siRNA and treated with iHDAC6. Scale bar: 20 μm.
(TIF)
