# Supplementary figures and images for: Histone deacetylase inhibition prevents cell death induced by loss of tricellular tight junction proteins in temperature-sensitive mouse cochlear cells
Source: PLoS One. 2017 Aug 2;12(8):e0182291. doi: 10.1371/journal.pone.0182291 (PMC5540400; doi:10.1371/journal.pone.0182291)

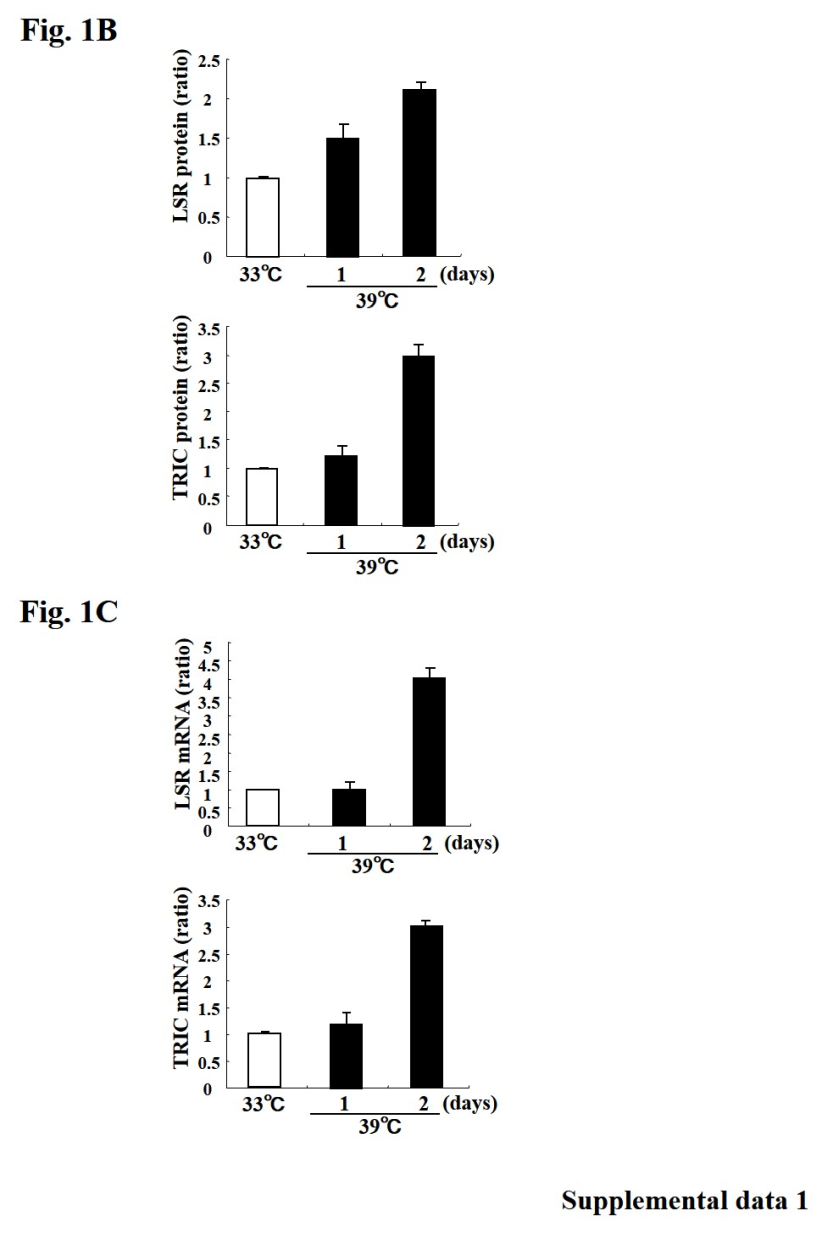

Supplement: S1 Fig — (TIF) [file pone.0182291.s001.tif]

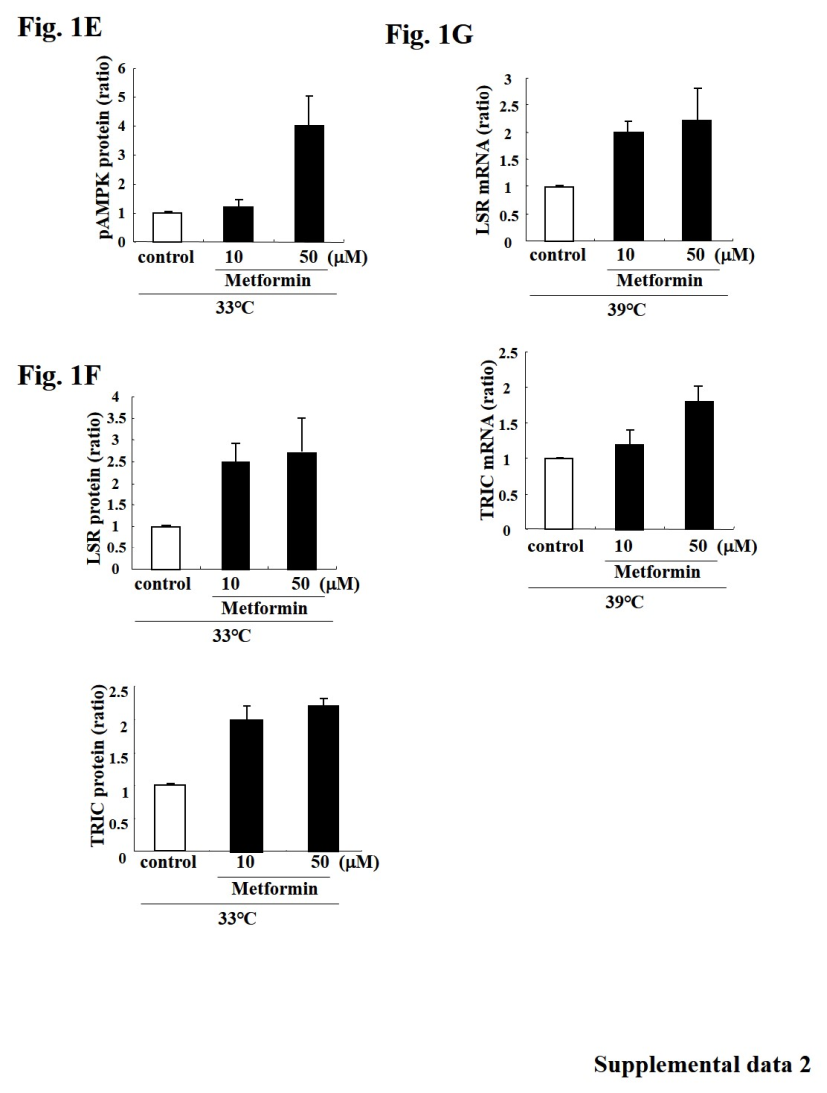

Supplement: S2 Fig — Graph of Fig 1 (E) western blotting for phospho-AMP kinase (pAMPK) in cells cultured at 33°C treated with 10 or 50 μM metformin. (F) Western blotting and (G) RT-PCR for LSR and TRIC expression in cells incubated at 39°C treated with 10 or 100 μM metformin. (TIF) [file pone.0182291.s002.tif]

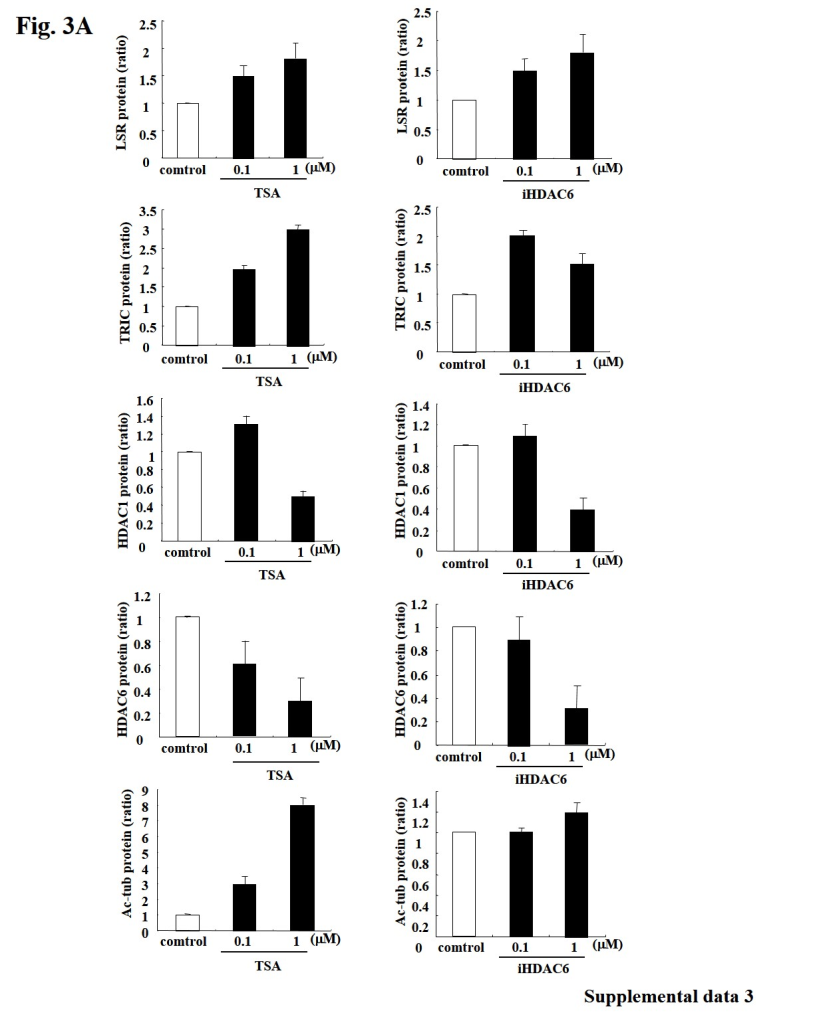

Supplement: S3 Fig — (TIF) [file pone.0182291.s003.tif]

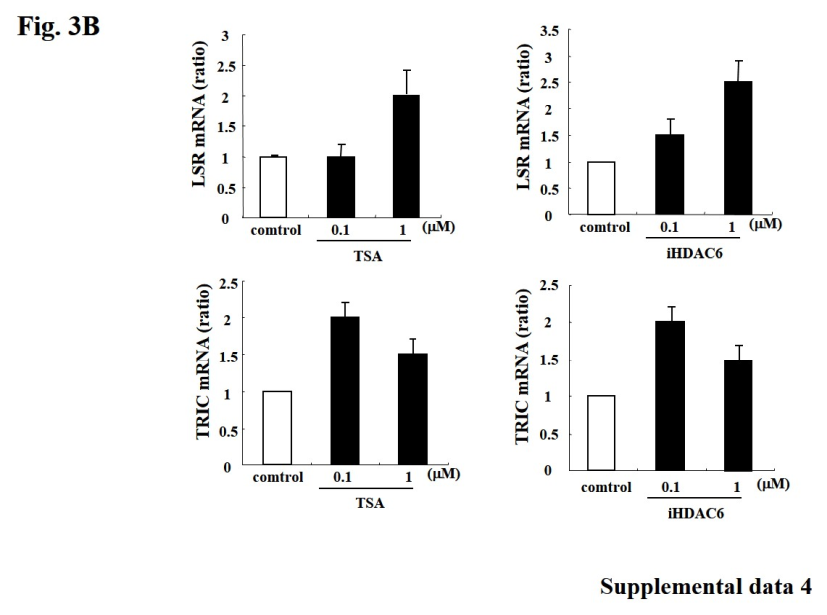

Supplement: S4 Fig — (TIF) [file pone.0182291.s004.tif]

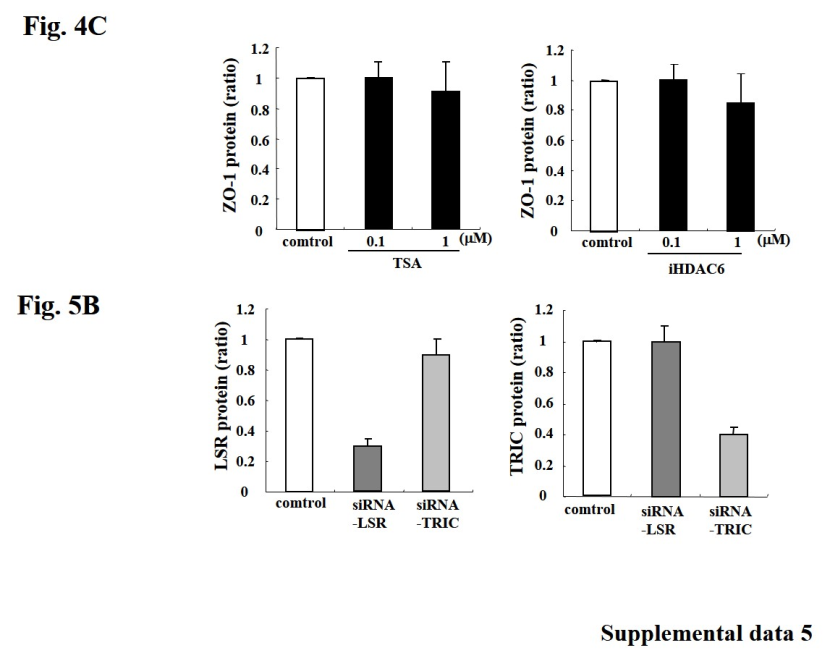

Supplement: S5 Fig — (TIF) [file pone.0182291.s005.tif]

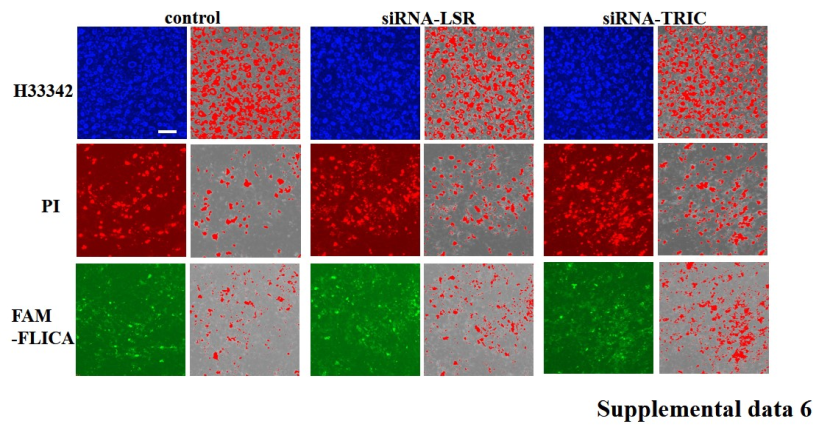

Supplement: S6 Fig — Scale bar: 20 μm. (TIF) [file pone.0182291.s006.tif]

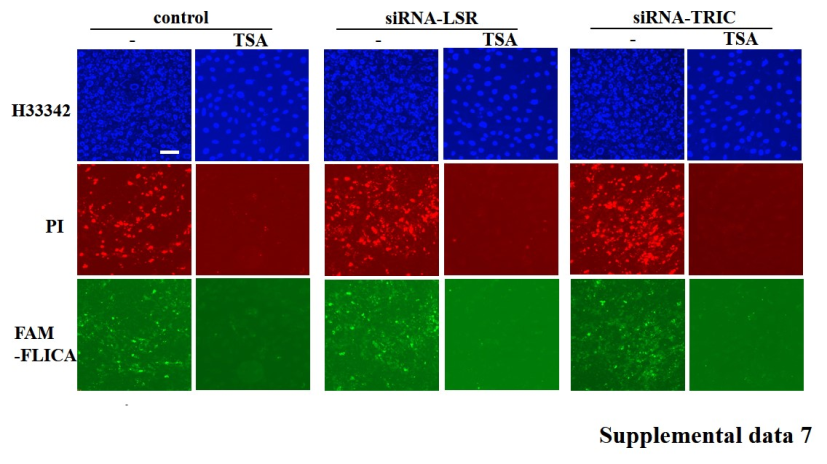

Supplement: S7 Fig — Scale bar: 20 μm. (TIF) [file pone.0182291.s007.tif]

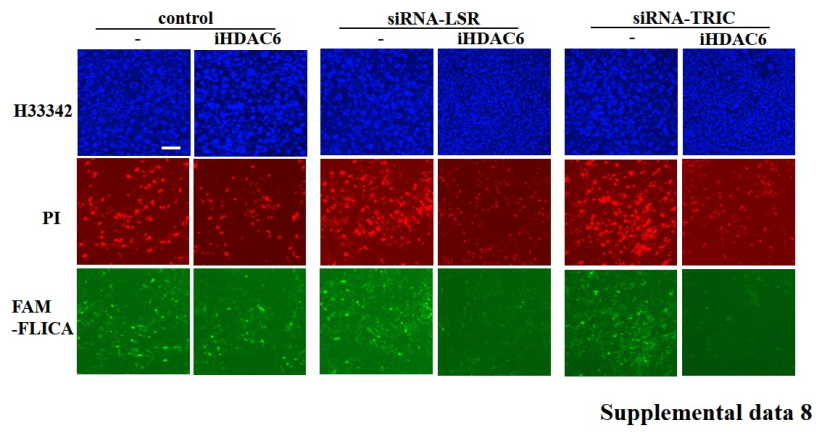

Supplement: S8 Fig — Scale bar: 20 μm. (TIF) [file pone.0182291.s008.tif]
